# Association of Fatigue Severity With Maladaptive Coping in Multiple Sclerosis: A Data-Driven Psychodynamic Perspective

**DOI:** 10.3389/fneur.2021.652177

**Published:** 2021-04-07

**Authors:** Gesa E. A. Pust, Jennifer Randerath, Lutz Goetzmann, Roland Weierstall, Michael Korzinski, Stefan M. Gold, Christian Dettmers, Barbara Ruettner, Roger Schmidt

**Affiliations:** ^1^Department of Psychology, University of Konstanz, Konstanz, Germany; ^2^Institut für Neuroimmunologie und Multiple Sklerose, Zentrum für Molekulare Neurobiologie Hamburg, Universitätsklinikum Hamburg-Eppendorf, Hamburg, Germany; ^3^Lurija Institute for Rehabilitation and Health Sciences at the University of Konstanz, Schmieder Foundation for Sciences and Research, Allensbach, Germany; ^4^Institute of Philosophy, Psychoanalysis and Cultural Studies, Berlin, Germany; ^5^Medical School Hamburg, Hamburg, Germany; ^6^Private Practitioner, London, United Kingdom; ^7^Charité — Universitätsmedizin Berlin, Klinik für Psychiatrie und Psychotherapie, Campus Benjamin Franklin, Berlin, Germany; ^8^Charité — Universitätsmedizin Berlin, Med. Klinik m.S. Psychosomatik, Campus Benjamin Franklin, Berlin, Germany; ^9^Klinik für Psychosomatik und Konsiliarpsychiatrie, Departement Innere Medizin, Kantonsspital St. Gallen, St. Gallen, Switzerland

**Keywords:** fatigue, multiple sclerosis, conversion, schema modes, latent profile analysis

## Abstract

Fatigue in persons with multiple sclerosis (PwMS) is severely disabling. However, the underlying mechanisms remain incompletely understood. Recent research suggests a link to early childhood adversities and psychological trait variables. In line with these studies, this paper took a psychodynamic perspective on MS-fatigue. It was hypothesized that fatigue could represent a manifestation of maladaptive coping with intense emotions. The schema therapeutic mode model served as a theoretical and empirically validated framework, linking psychodynamic theory and empirical research methods. The study was based on a data set of *N* = 571 PwMS that has also served as the basis for another publication. Data was collected online. The Schema Mode Inventory was used to quantify regulatory strategies to cope with emotionally stressful experiences. In addition, depressive symptoms (Beck's Depression Inventory - FastScreen), physical disability (Patient Determined Disease Steps), alexithymia (Toronto Alexithymia Scale-26), adverse childhood experiences (Childhood Trauma Questionnaire), and self-reported fatigue (Fatigue Scale for Motor and Cognitive Functions) were assessed. Latent profile analysis revealed three distinct groups of PwMS, based on their coping mode profiles: (1) PwMS with low maladaptive coping, (2) PwMS with avoidant/submissive coping styles, and (3) PwMS with avoidant/overcompensatory coping styles. Multivariate comparisons showed no significant difference in physical disability across the three groups. However, heightened levels of self-reported fatigue and depression symptoms occurred in PwMS with maladaptive coping styles. A path model uncovered that self-reported fatigue was robustly related to physical disability (β = 0.33) and detached/avoidant coping (Detached Protector; β = 0.34). There was no specific relation between any of the maladaptive coping modes and depression symptoms. Detached/avoidant coping was in turn predicted by childhood emotional abuse and neglect. The results indicate that childhood adversity and detached/avoidant coping styles may be associated with variability in MS-fatigue severity: PwMS that resort to detached/avoidant coping in response to negative emotions also tend to report heightened levels of fatigue, although they do not differ in their perceived disability from PwMS with low levels of fatigue and maladaptive coping. A link between MS-fatigue and the psychodynamic traumatic conversion model is discussed. The implications of these findings for therapeutic interventions require further study.

## Introduction

Fatigue is among the most disabling symptoms in multiple sclerosis (1). The Multiple Sclerosis Council for Clinical Practice Guidelines (2) defines fatigue as “a subjective lack of physical and/or mental energy that is perceived by the individual or caregiver to interfere with usual or desired activities.” The symptoms range from difficulties in writing and walking to a total loss of work ability.

To date, research has implicated various factors that might contribute to the development of fatigue in persons with multiple sclerosis (PwMS). These may include neurodegenerative and inflammatory processes, or life-style associated risks, such as diet or substance use (3, 4). Integrative etiological models have conceptualized MS-fatigue as an interplay of biological, psychological, and social factors (5–7). Still, its multifactorial pathogenesis remains incompletely understood. While meta-analyses have supported the efficacy of both pharmacological (8) and behavioral interventions, most notably cognitive behavioral therapy (CBT) (9), evidence-based treatment recommendations remain scarce (10). This still poses a challenge to clinical practice in the search for effective treatments to tackle the immense burden of MS-fatigue.

Fatigue in general is among those symptoms that often cannot sufficiently be explained by physical factors (11). This calls for studies, which also attempt to understand the functional nature of MS-fatigue from a non-somatic perspective, in particular focusing on psychological factors. Latest studies have uncovered a relation between early childhood adversities and particular trait characteristics with MS-fatigue (12, 13). In our previous publication based on this sample (12), a specific pattern among the five childhood adversities subscales taken from the Childhood Trauma Questionnaire (14, 15), and their association with specific trait characteristics and MS-fatigue could be identified: PwMS who reported emotional neglect and emotional abuse demonstrated impairments in autonomy and performance as well as high rates of alexithymia. These factors in turn predicted fatigue severity. The results suggest a significant contribution of psychological/psychosomatic factors on the development of MS-fatigue, in terms of fatigue as a manifestation of maladaptive processes that find its origin in early adversities. The view that MS-fatigue could be associated with maladaptive psychological processes is supported by previous studies demonstrating that PwMS with fatigue tend to react with avoidance to fearful stimuli (6, 7).

Based on the previous findings, the present study hypothesized that early adversities manifest psychopathological processes that in turn result in the development or amplification of MS-fatigue. It puts is emphasis on the psychosomatic etiology and functional nature on MS-fatigue as a bodily symptom with a psychological origin. This study is rooted in psychodynamic models for the development of conversion disorders. To combine this theoretical framework with quantitative research methods, it utilized a modern integrative and empirical approach (schema therapy).

From a psychodynamic perspective, MS-fatigue *per se* is not considered as a conversion disorder in the classical, i.e., conflictual sense. Nevertheless, the available research on the association with early adversities in-deed suggests that a psychogenic traumatic conversion perspective might apply to MS-fatigue too. In line with the current DSM-5 definition (16), a conversion disorder can be defined as the occurrence of neurological-like symptoms that cannot fully be explained by organic pathology. This matches with results from studies demonstrating that physical factors alone are not sufficient to explain MS-fatigue (11). These deficits are neither intentionally produced nor feigned (17). Since the early theories, conversion disorder has always been considered as the consequence to a painful or stressful event (18), even if this strict causality is no longer part of the DSM diagnostic criteria. The psychodynamic perspective on psychosomatic symptoms proposes distinct and refined theoretical approaches:

- According to ***Janet's dissociation model***, an individual can develop unspecific bodily symptoms as a consequence to dissociated negative and unbearable emotions (19). This means, that an individual would neutralize parts of the (traumatic) emotions and resort to the defense mechanisms of “splitting” and “disavowal” (20), in order to deal with the fearful, traumatic reality.
- In line with ***Freud's trauma model*** that finds its origins in Janet's work, the actual neurosis lies in the inability of transforming emotional excitation into mental phenomena and discharging it into the body instead (21). This means, that the individual, who is exposed to a traumatic event, resorts to splitting off the trauma-related excitations as an unconscious defense mechanism. Consequently, the overwhelming fear-related affects are not consciously perceived and processed. Instead, the potentially trauma related emotional exhaustion manifests in fatigue as a bodily symptom. According to the trauma model, fatigue in terms of neurasthenia represents an ***asymbolic form*** ***of conversion***. Especially alexithymic patients would perceive the physiological aspect of their emotions, while not being able to recognize or name the psychological quality of these emotions (22).
- Contrary to the asymbolic conversion referred to in the trauma model, ***Freud's psychoneurotic conversion model*** proposes a symbolism of the bodily symptoms (20): A phantasy accompanied by reluctance is pushed into the unconscious, and replaced by a bodily symptom (fatigue). This type of “hysterical somatization” represents failed repression so that the repressed returns in form of the bodily symptom, for example as fatigue (23). The classic work by Franz Alexander and Franz Deutsch also proposed a symbolic specificity, i.e., that specific emotions and personality types correspond to specific types of physical dysfunction (24, 25).

In line with the studies that have identified early adversities and avoidance of fearful stimuli being associated with MS-fatigue, the present study selected the trauma model as its theoretical basis. It explored MS-fatigue in relation to general coping mechanisms occurring as a response to negative and fearful affects. It aimed to understand if PwMS with fatigue indeed react with avoidance to adverse affects, as a manifestation of the individual's defense mechanisms proposed in the trauma model. It has also to be taken into account that to the best of our knowledge no research exists yet that would allow generating testable hypotheses for a specific relation between MS-fatigue and dissociation on the one hand, or MS-fatigue and unconscious phenomena on the other. Thus, the trauma model treating fatigue as an asymbolic converse symptom was selected as the basis for the present study. The other two theoretical perspectives on MS-fatigue were discarded in this study.

In order to resort to a theoretical and empirically validated framework that allows distinguishing stable coping responses to adverse overwhelming emotional states, the schema therapeutic mode model was utilized (26). Schema therapy that also resorts to psychodynamic perspectives was applied to the present research question. It was initially developed to treat chronic maladaptive response patterns (so-called “schemas”), in particular in individuals suffering from personality disorders (27). It is proposed that individuals respond to adverse affects with recurrent coping patterns (so-called “maladaptive coping modes”) in terms of fight (overcompensation), flight/faint (avoidance), or surrender. These maladaptive coping modes correspond to universal human coping mechanisms (28). They are thought to originate from adverse childhood experiences and the frustration of core needs. These early adversities also include trauma exposure, i.e., events that elicit highly negative affect (27), where the development of maladaptive coping modes served to prevent the individual from overwhelming negative affects. Maladaptive coping modes therefore represent the conscious and visible response to underlying defense mechanisms. Early coping mechanisms become maladaptive when they are inflexible and stable, and prevent the individual from perceiving and processing potential future situations in an appropriate way.

For the present study, schema therapy in connection with the traumatic conversion model therefore provides an integrative theoretical framework to investigate processes that might occur within the trauma model by means of empirical methods. This study was conducted with a large and representative sample of PwMS utilizing a data-driven approach. It was hypothesized that (1) MS-fatigue is a phenomenon that is related to the recruitment of specific avoidance-associated coping mechanisms, and (2) that these coping mechanisms are in turn related to specific early adversities. In addition, it was investigated if the recruitment of specific coping mechanisms is also associated with alexithymia due to the high relevance of alexithymia in psychodynamic theories on conversion.

## Materials and Methods

### Participants

The study was advertised with the following methods: (1) a link on the website of the German MS Society (Deutsche Multiple Sklerose Gesellschaft, DMSG) served to allow direct access to the online survey (Unipark survey software, Globalpark AG, Hürth); (2) flyers and newsletters were distributed at a large rehabilitation center (Kliniken Schmieder Konstanz) and the MS outpatient clinic of the University Medical Center Hamburg-Eppendorf. The flyers provided a link to connect to the survey. Sampling took part from July 2018 to March 2019. Patients with a self-reported diagnosis of MS and an age of at least 18 years were eligible to participate. A total of 1,490 individuals registered via the website. 608 (41 %) PwMS completed the study. **Table 3** in the results section provides a detailed overview on the socio-demographic and self-reported clinical data of the PwMS for the total sample, as well as the sub-groups derived from the subsequent analyses. To avoid bias due to the imputation of missing data, only full data sets of 571 PwMS were included for the final analyses. Prior to enrolment, all participants provided full informed consent. The ethical review board of the University of Konstanz approved the study (“BESTÄTIGUNG 6/2018”). The study was conducted in line with the latest European data protection regulation (EU-GDPR).

### Study Procedure

Before access to the survey was granted, PwMS had to give their informed consent by clicking the “agree” button referring to the terms of participation. After accepting the respective terms and conditions, PwMS were directed to the survey. In a first step, relevant patient characteristics were assessed (sex, age, MS duration, disease course, medication, education, family status, psychiatric disorders), and Patient Determined Disease Steps [PDDS; (29)] as a measure of disability. For keeping the survey short, only the most relevant patient characteristics were assessed. Second, PwMS had to respond to questions from a set of specific questionnaires (see below). Completing the whole survey did not take longer than ~65 min. The data gathered in this study relies solely on the PwMS' input. No specific online survey guidelines were considered.

### Assessment of Fatigue Severity

The study included two common measures of fatigue, (1) the Fatigue Scale for Motor and Cognitive Functions [FSMC; (30)], and (2) the Chalder Fatigue Questionnaire [CFQ; (31)].

The FSMC comprises 20-items. Ten items serve to measure symptoms of motor and cognitive fatigue, respectively (30). For each item, a five-point Likert scale is provided (from “1” = absolutely agree” to “5” = “absolutely disagree”). In the present study, the total score, which is the sum of the 20 individuals item scores (*FSMC*; range: 20–100) was utilized. Based on a recent study in a large sample of 1.403 PwMS that investigated the factorial validity of the FSMC and failed to confirm the validity of the distinction between the two sub-constructs of motor and cognitive fatigue (32), only the sum score of the fatigue scale was considered.

Another well-established measure to assess patients' fatigue severity is the CFQ (31). Eleven items refer to symptom severity of fatigue in the past 4 weeks. A four-point Likert scale (“0” = “less than usual,” to “3” “much more than usual”) is provided for the scoring of the responses. For the present study, the German version was administered (33). As for the FSMC and in line with the study of Pust and colleagues (32), only the CFQ global severity score was used and calculated as the sum of all items (*CFQ*; range: 0–33).

### Assessment of Depression Symptoms

The present study made use of the German version of the Beck Depression Inventory [*BDI-II*, (34)] as a measure to assess current symptoms of depression. The BDI comprises 21 items. The symptom severity for each item can vary between “0” and “3.” For each symptom, an individual description of its potential manifestation is provided. Higher values indicate a higher symptom severity. The total score for depression ranges from 0 to 63. According to the German “Nationale Versorgungs Leitlinie Unipolare Depression,” the official evidence- and consensus-based clinical practice guidelines (35), score above 13 indicate clinically relevant depressive symptoms. In the subsequent analyses, only the seven-item BDI-II-FastScreen score [*BDI-FastScreen*; (36)] was included. The latter one omits items covering vegetative and somatic aspects of depression and thus does not overlap with fatigue. It is the sum of seven non-somatic depression items (e.g., dysphoria, anhedonia, suicidal ideation; range: 0–21).

### Assessment of Disability

The current study made use of the German version of the Patient Determined Disease Steps (*PDDS*) a measure of disability. A nine-point scale is provided (“0” = “normal” to “8” = “bedridden”) (29). Higher scores indicate a higher disability. The PDDS serves as a self-report measure but has demonstrated strong correlations with the neurologist-rated Expanded Disability Status Scale [EDSS; (37)], proving its validity.

### Assessment of Alexithymia

For the assessment of alexithymia, the German version of the Toronto-Alexithymia-Scale-26 (*TAS-26*) developed by Kupfer et al. (38) was administered. The TAS-26 is a reliable and valid self-report scale comprising 26 items. Each item is rated on a five-point Likert scale (“1” = “not at all true” to “5” = “absolutely true”). The total alexithymia score is the sum of responses to 18 of the 26 items (range: 18–90).

### Assessment of Childhood Adversities

For the assessment of childhood adversities, the present study made use of the reliable and valid German short-version (15) of the Childhood Trauma Questionnaire [CTQ; (14)]. The CTQ is a self-report questionnaire and contained 28 items in the present study. The CTQ covers the exposure to different types of childhood adversities. A five-point Likert scale serves to indicate the frequency of exposure to the various adversity types (“1” = “never true”; “5” = “very often true”). Five subscales are distinguished: 1. *CTQ emotional abuse*; 2. *CTQ physical abuse*; 3. *CTQ sexual abuse*; 4. *CTQ emotional neglect*, and 5. *CTQ physical neglect*. For the sum score of each subscale, each of the five item scores belonging to one subscale are summed. Subscores thus can range from 5 to 25. The present study did not consider the additional three-item scale on social desirability.

### Assessment of Maladaptive Coping Modes

As a measure for the PwMS' maladaptive coping modes, the German version of the Schema Mode Inventory [SMI; (39)] was administered. The 118-item questionnaire assesses 18 different modes according to Young's schema mode model. For every item, participants have to rate on a six-point Likert scale, as to which extend the items apply to them (0 = “never or almost never” to 6 = “always”). For the present study, only the 39 items belonging to the maladaptive coping modes were considered. The 39 items can be assigned to the three individual responses to psychic stress in terms of *Surrender, Avoidance* and *Overcompensation*, whereas each of the latter two contains two distinct modes [*Avoidance*: (1) *Detached Protector* (passive avoidance mode), and (2) *Detached Self-Soother* (active avoidance mode); *Overcompensation*: (1) *Self-Aggrandizer*, and (2) *Bully and Attack mode*]. For the calculation of the subscores for the five maladaptive coping modes, item scores can be summed (*SMI Compliant surrender*: 7 items; *SMI Detached Protector*: 9 items; *SMI Detached Self Soother*: 4 items; *SMI Self Aggrandizer*: 10 items; *SMI Bully and Attack mode*: 9 items). To facilitate the comparison between the different coping mode scores, mean scale scores (range 0–6) are reported in the subsequent analyses.

### Data Analysis

A four-step process was chosen to analyse the data. First, latent profile analysis (LPA) (40) was utilized to identify subgroups of PwMS response patterns to distress, as measured by the SMI's dysfunctional coping modes items. LPA allows to account for different response patterns in the modes avoidance, surrender and overcompensation simultaneously. LPA was conducted using Mplus Version 7 for Mac. LPA uses latent categorical variables to identify groups of individuals (classes) with similar response patterns on a set of manifest variables, i.e., the maladaptive coping modes items. In comparison to other statistical approaches that aim to identify groups of participants within a dataset, LPA provides the availability of more rigorous empirical criteria for determining the number of clusters by combining different fit indicators (41). Due to the positively skewed and non-normally distributed data in the SMI items, a Poisson model was preferred over a linear model for continuous data. As the items' standard deviations did not exceed the items' means, the application of a negative binomial model was discarded. However, rerunning the analyses with a negative binomial did not lead to different results. Applying a zero-inflated negative binomial model to the data was discarded due to unacceptable fit indices. Latent profile analyses were calculated for two- to eight-classes models. Poisson models with 100 random sets of starting values for the initial stage and 50 final stage optimizations were selected. For the selection of the appropriate number of classes, indicating the superiority of the final model in comparison to models with a different number of classes, Lo–Mendell–Rubin-adjusted likelihood ratio tests (LMR-A) as well as bootstrap likelihood ratio test (BLRT) were calculated (40). The Bayesian Information Criterion (BIC) was chosen as a model fit indicator. To test for differences in the socio-demographic and clinical variables across the three LPA groups, one-way analyses of variance (ANOVAs) as well as Chi-Square-tests were calculated. As a more conservative approach, no alpha-adjustment was conducted.

Next, after identifying the most appropriate number of classes, ANOVAs were conducted with class membership as the independent variable and mean scores in the SMI modes *Compliant Surrender, Detached Protector, Detached Self-Soother, Self-Aggrandizer* and *Bully and Attack mode* as dependent variables. This step served to characterize the coping mode profiles of the PwMS in the different classes. To test for differences between class membership in the PwMS' depression and fatigue symptoms as well as the level of disability, further ANOVAs were calculated. Since *F-*tests are robust to non-normal distributions (42), tests against normality of the distribution were not performed. Tamhane t2 *post-hoc* tests were calculated to account for unequal variances across classes. Tamhane takes into account inhomogeneous variances combined with an α-correction according to Sidak. Analyses were conducted using R statistics and SPSS 27, applying a cut-off level for significance of *p* < 0.05.

Third, to analyse the relation between the PwMS' maladaptive coping modes and their depression as well as fatigue symptoms, a multivariate linear regression model was applied, using AMOS 24 for MAC. The level of disability served as a co-variate. Non-significant paths were removed in a backward exclusion procedure. Cut-off criteria to evaluate the model fit were defined according to Hu and Bentler (43), considering the thresholds for an at least acceptable fit: CFI (Comparative Fit Index) ≥ 0.95; SRMR (Standardized Root Mean Square Residual) < 0.10; NNFI (Nonnormed Fit Index) ≥ 0.95; RMSEA (Root Mean Square Error of Approximation) ≤ 0.08. Finally and *post-hoc*, a linear regression model was calculated for the prediction of the Detached Protector mode by childhood maltreatment and alexithymia.

## Results

### Class Assignment by Symptom Profiles and Differences in Socio-Demographic and Clinical Variables Across LPA-Groups

For the two- and three-class model of the LPA, LMR-A and BLRT tests were significant on a *p* < 0.05 level. For the comparison between the three- and four classes model, the LMR-A test did not reach statistical significance. Consequently, the three-class solution was selected. Considering all available fit measures, the three classes model (Cluster 1: *n* = 301; Cluster 2: *n* = 194; Cluster 3: *n* = 76) fulfilled all recommendations for a fitting model defined by Nylund and colleagues (40). Table 1 gives an overview of the seven different models' fit indices.

**Table 1:** Fit indices for the seven different latent profile analyses.

**Modell**	**Log-likelihood**	**BIC**	**Entropy**	**LMR-A *p***	**BLRT *p***
2 classes	−35,177.48	70,856.34	0.81	<.001	<.001
**3 classes**	–**35,067.94**	**70,891.22**	**0.80**	**0.025**	**0.025**
4 classes	−35,050.86	71,110.94	0.83	0.172	0.171
5 classes	−34,978.79	71,220.71	0.76	0.267	0.266
6 classes	−34,937.86	71,392.75	0.79	0.194	0.194
7 classes	−34,918.37	71,607.65	0.82	0.526	0.526
8 classes	−34,901.33	71,827.48	0.81	0.531	0.531

*Bayesian information criterion (BIC), Lo-Mendell-Rubin adjusted likelihood ratio test (LMRA-A); bootstrap likelihood ratio test (BLRT). Fit indices for the selected 3 classes model are printed bold*.

**Figure 1:**
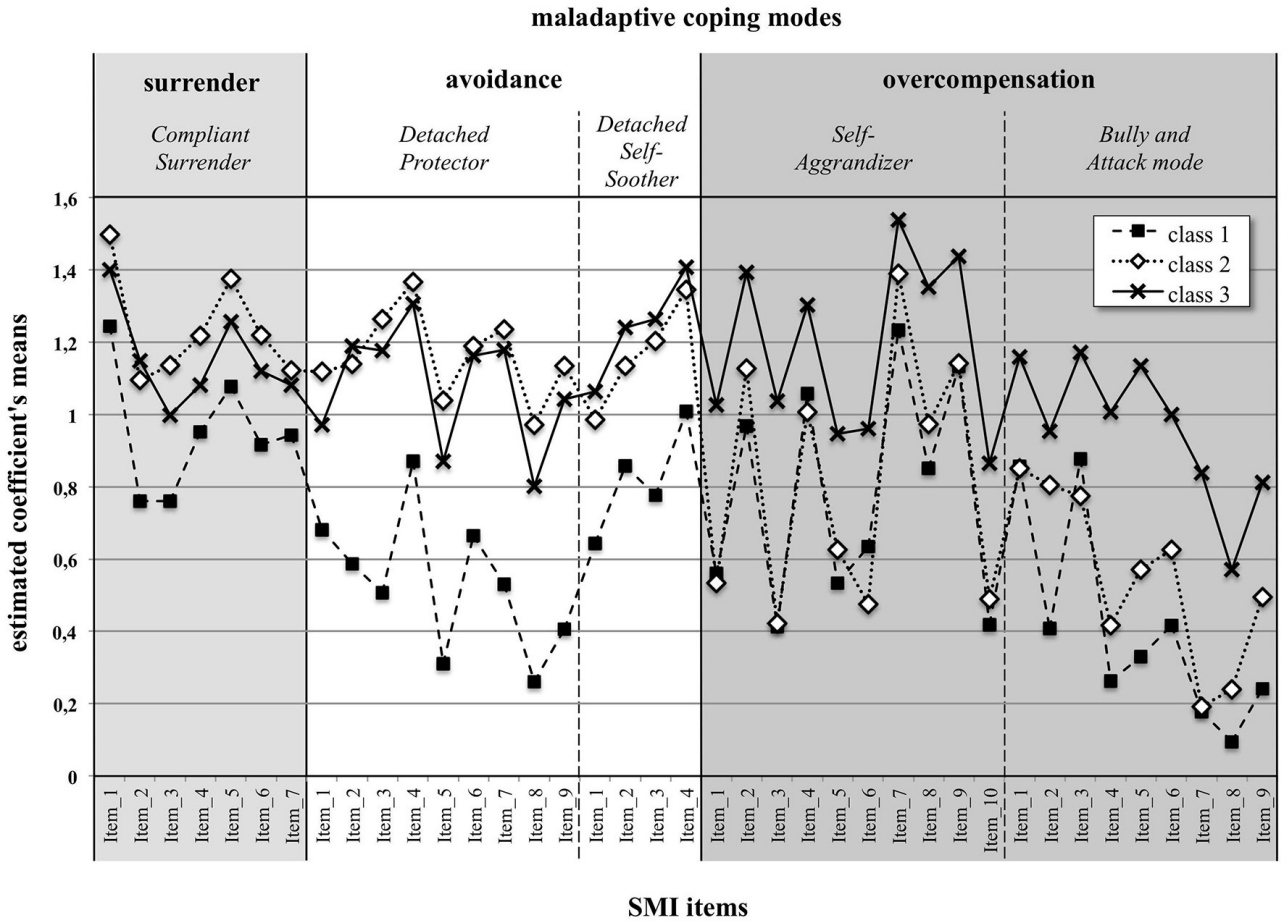
Item response profiles of the three classes of PwMS in the five SMI maladaptive coping modes. The items belonging to the coping strategy of surrender are colored in light gray. The items belonging to avoidance (Detached Protector and Detached Self-Soother) are colored white. Colored in dark gray are those items belonging to overcompensation, i.e., the Self-Aggrandizer mode and the Bully and Attack mode. The estimated means were derived from the previous LPA.

The mean response profiles in the SMI dysfunctional coping modes items for the three classes are displayed in Figure 1.

Results in Table 2 indicate the number of cases in each of the three classes. The posterior probabilities that participants belonged to their assigned class ranged between 0.81 and 0.95. Only for the third class, some PwMS could not unequivocally be assigned to that class and had some overlap in their response profile with the second class. Still, the model selection produced a meaningful class assignment with three distinguishable classes, as also supported by the entropy value of 0.80 [Table 1; see (44) for statistical details].

**Table 2:** Average posterior probabilities for the 3-class model.

**class**	***N***	**1**	**2**	**3**
class 1	301	**0.95**	0.04	0.01
class 2	194	0.07	**0.89**	0.03
class 3	76	0.04	0.15	**0.81**

*Posterior probabilities represent the probability that an individual belongs to the respective assigned class. Bold values represent the average probabilities of the corresponding classes*.

Table 3 displays the descriptive statistic for the socio-demographic and clinical variables, (1) for the total sample, as well as (2) for the three LPA classes. There were no statistically significant differences across the three classes, except for the variable “highest degree” and “current psychiatric disorder.” For the latter variable, no difference was obtained between classes 2 and 3.

**Table 3:** Socio-demographic and clinical data (1) of the total sample, (2) and separated by LPA classes.

		**LPA class**			
**Measure**	**Total sample**	**class 1**	**class 2**	**class 3**	**Statistics**
	**(*n* = 571)**	**(*n* = 301)**	**(*n* = 194)**	**(*n* = 76)**	
**Sex** ***n*** **(%)**					
Female	438 (76.7)	238 (79.1)	149 (76.8)	51 (67.1)	Chi^2^(2) = 4.86, *p* = 0.088
Male	133 (23.3)	63 (20.9)	45 (23.2)	25 (32.9)	
**Age** (*M* ±*SD*)	43.4 (10.9)	44.0 (11.1)	42.8 (10.9)	42.5 (9.7)	*F*_(2, 568)_ = 0.96, *p* = 0.385
**Disease duration** in years (*M* ±*SD*)	9.1 (7.6)	9.7 (8.1)	8.4 (7.2)	8.4 (7.0)	*F*_(2, 568)_ = 2.21, *p* = 0.111
**Medication** ***n*** **(%)**					
Yes	399 (69.9)	211 (70.1)	139 (71.6)	49 (64.5)	Chi^2^(2) = 1.35, *p* = 0.509
No	172 (30.1)	90 (29.9)	55 (28.4)	27 (35.5)	
**Disease course** ***n*** **(%)**					
Primary manifestation	7 (1.2)	3 (1.0)	3 (1.5)	1 (1.3)	Chi*^2^*(8) = 5.42, *p* = 0.712
RRMS	392 (68.7)	210 (69.8)	130 (67.0)	52 (68.4)	
PPMS	52 (9.1)	22 (7.3)	23 (11.9)	7 (9.2)	
SPMS	75 (13.1)	45 (15.0)	21 (10.8)	9 (11.8)	
Not specified	45 (7.9)	21 (7.0)	17 (8.8)	7 (9.2)	
**Highest school degree**					
No school degree	2 (0.4)	0 (0.0)	1 (0.5)	1 (1.3)	Chi^2^(6) = 12.15, *p* = 0.059
Lower-secondary qualification (Hauptschulabschluss)	39 (6.8)	20 (6.6)	11 (5.7)	8 (10.5)	
Intermediate general qualification (Mittlere Reife)	173 (30.3)	87 (28.9)	71 (36.6)	15 (19.7)	
University entrance qualification (Abitur)	357 (62.5)	194 (64.5)	111 (57.2)	52 (68.4)	
**Highest degree**					
No vocational training	25 (4.4)	12 (4.0)	9 (4.6)	4 (5.3)	Chi^2^(6) = 19.02, *p* = 0.004
Vocational training (Lehre)	246 (43.1)	117 (38.9)	103 (53.1)	26 (34.2)	
Technical school (Fachschule)	97 (17.0)	49 (16.3)	27 (13.9)	21 (27.6)	
University degree/polytechnic degree	203 (35.6)	123 (40.9)	55 (28.4)	25 (32.9)	
**Family status**					
Married, living together	274 (48.0)	151 (50.2)	84 (43.3)	39 (51.3)	Chi^2^(12) = 16.13, *p* = 0.185
Married, living apart	11 (1.9)	5 (1.7)	3 (1.5)	3 (3.9)	
Same-sex union, living together	2 (0.4)	2 (0.7)	0 (0.0)	0 (0.0)	
Cohabiting	116 (20.3)	63 (20.9)	38 (19.6)	15 (19.7)	
Divorced	21 (3.7)	15 (5.0)	4 (2.1)	2 (2.6)	
Widowed	7 (1.2)	3 (1.0)	4 (2.1)	0 (0.0)	
Single	140 (24.5)	62 (20.6)	61 (31.4)	17 (22.4)	
**Current psychiatric disorder** ***n*** **(%)**					
Yes	164 (28.7)	55 (18.3)	77 (39.7)	32 (42.1)	Chi^2^(2) = 34.11, *p* < 0.001 (Chi^2^ (1) = 0.13, *p* = 0.716)
No	407 (71.3)	246 (81.7)	117 (60.3)	44 (57.9)	

*RRMS, relapsing–remitting multiple sclerosis; PPMS, primary progressive multiple sclerosis; SPMS, secondary progressive multiple sclerosis. Test statistics refer to the comparisons between the three LPA classes. For the variable “current psychiatric disorder,” test statistics in brackets refer to the comparison of class 2 and 3 only*.

### Differences in Maladaptive Coping Modes Across Classes

**Figure 2:**
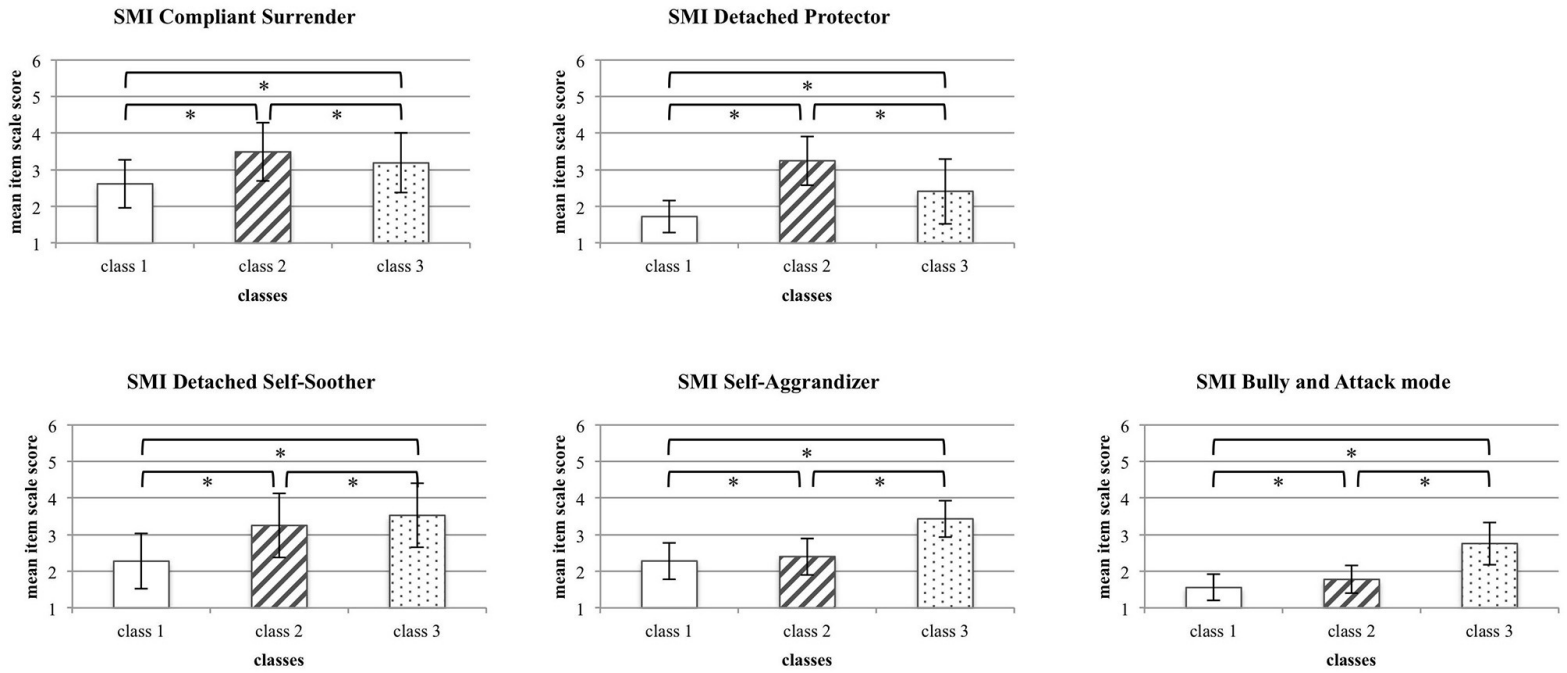
Differences in maladaptive coping modes between the three classes. For each coping mode, mean item scores (±SD) are displayed. Asterisks highlight statistically significant differences across classes.

In a second step (for graphic illustration, see Figure 2), mean differences between the three classes were compared using one-way ANOVAs for the five SMI dysfunctional coping modes: (1) Compliant Surrender [*F*_(2, 569)_ = 86.33, *p* < 0.001, $\eta_{\text{p}}^{2}$ = 0.23], (2) Detached Protector [*F*_(2, 569)_ = 403.70, *p* < 0.001, $\eta_{\text{p}}^{2}$ = 0.59], (3) Detached Self-Soother [*F*_(2, 569)_ = 118.87, *p* < 0.001, $\eta_{\text{p}}^{2}$ = 0.30], (4) Self-Aggrandizer [*F*_(2, 569)_ = 161.63, *p* < 0.001, $\eta_{\text{p}}^{2}$ = 0.36], and (5) Bully and Attack mode [*F*_(2, 569)_ = 264.79, *p* < 0.001 $\eta_{\text{p}}^{2}$ = 0.48].

All *post-hoc* Tamhane t2 tests for pairwise comparisons were also statistically significant, except for the difference between the mean SMI Detached Self-Soother score of class 2 and class 3 (*p* = 0.071), i.e., the actively avoidant coping mode. However, when taking the obtained effect sizes into account, only (1) the pronounced Surrender coping in class 2, (2) the pronounced Overcompensation coping in class 3, and (3) the pronounced Avoidance coping in both classes 2 and 3 compared to class 1 could be considered as meaningful.

Thus, the results revealed three classes with distinct symptom profiles. Class 1 (*Low overall dysfunctional coping*) had the lowest scores in all SMI maladaptive coping modes compared to the other two classes, PwMS in class 2 (*Avoidance/Surrender coping*) rather demonstrate maladaptive coping in terms of Surrender and Avoidance, whereas class 3 (*Avoidance/Overcompensation*) tends to demonstrate dysfunctional coping in terms of Overcompensation and Avoidance.

**Figure 3:**
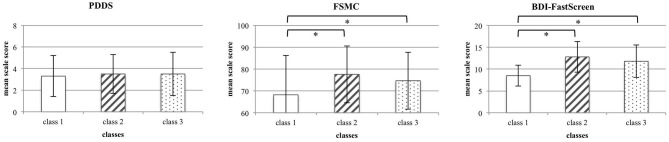
Differences in disability, fatigue and depression symptoms between the three classes. For each measure, mean total scores (±SD) are displayed. Asterisks highlight statistically significant differences across classes.

There was no difference in the disability between the three classes [mean class 1 (*SD*): 2.3 (1.9); mean class 2 (*SD*): 2.5 (1.8); mean class 3 (*SD*): 2.5 (2.0); F_(2, 569)_ = 0.82, *p* < 0.442, $\eta_{\text{p}}^{2}$ = 0.03]. The fatigue severity as well as the depression severity differed between the three groups [FSMC: mean class 1 (*SD*): 68.2 (17.6); mean class 2 (*SD*): 77.6 (13.0); mean class 3 (*SD*): 74.7 (13.2); *F*_(2, 569)_ = 22.32, *p* < 0.001, $\eta_{\text{p}}^{2}$ = 0.07; BDI-FastScreen: mean class 1 (*SD*): 2.5 (2.4); mean class 2 (*SD*): 6.8 (3.5); mean class 3 (*SD*): 5.8 (3.7); *F*_(2, 569)_ = 124.49, *p* < 0.001, $\eta_{\text{p}}^{2}$ = 0.31], however, the *post-hoc* calculated Tamhane t2 tests only revealed higher symptoms in classes 2 and 3, but no differential symptom pattern between the latter two groups (see Figure 3).

Thus, PwMS with maladaptive coping mechanisms in relation to adverse emotional states reported more severe fatigue symptoms. However, a specific association between fatigue symptoms and either more pronounced Surrender or Overcompensation was not yet supported. Consequently, a path model was calculated for the specific association between the SMI dysfunctional coping modes and fatigue as well depression symptoms.

### Predicting Fatigue and Depression Symptoms From Disability and Dysfunctional Coping Modes

**Figure 4:**
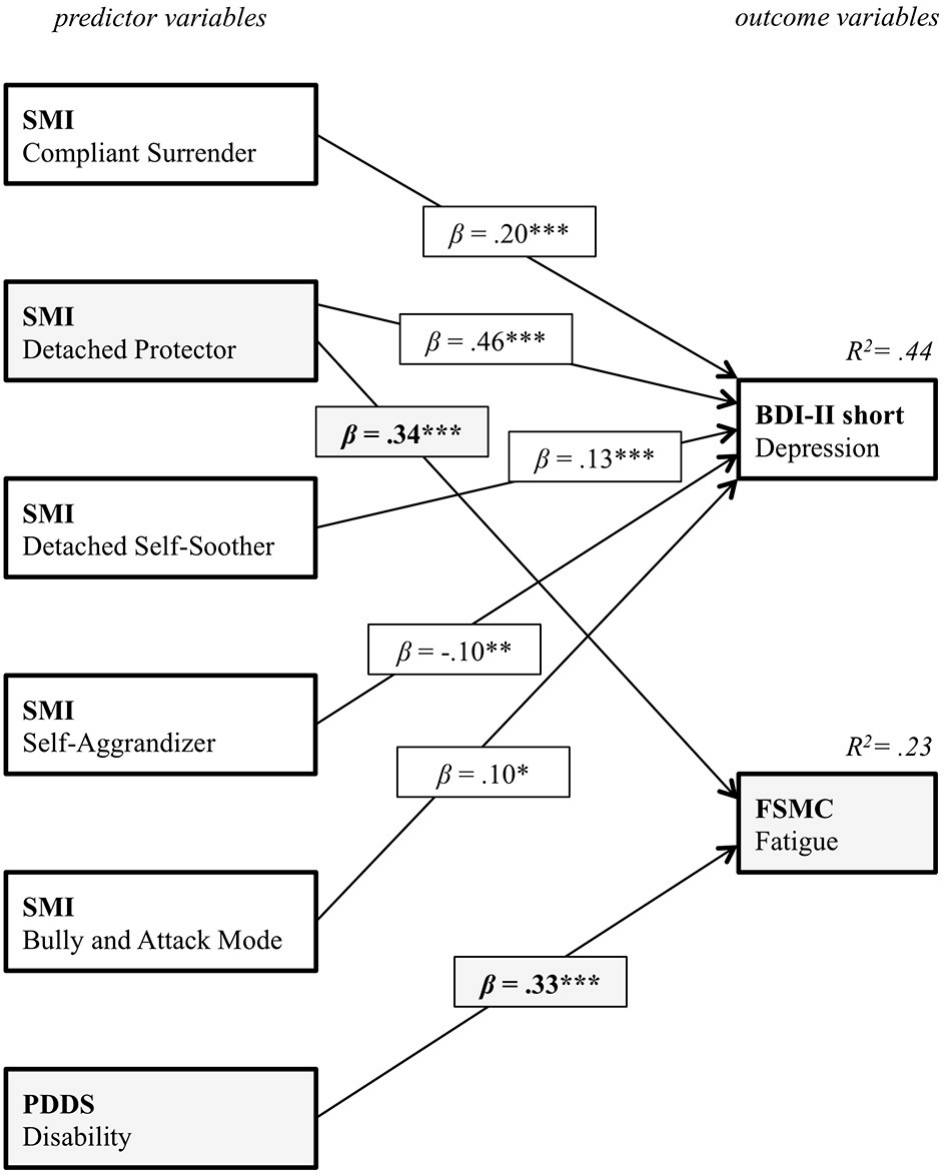
Pathmodel for the relation between the five SMI maladaptive coping modes, the PwMS' disability and the fatigue as well as depression symptoms.

A path model was calculated with the five SMI maladaptive coping modes and disability as predictors as well as the PwMS' depression and fatigue symptoms as dependent variables. The aim was to identify specific relations between maladaptive coping modes and depressions as well as fatigue symptoms, disentangling the relative contribution of the different modes to depression and fatigue symptoms. The final path model after stepwise removal of insignificant paths, in order to improve model fit, is displayed in Figure 4. For a better visual representation, the beta coefficients for the two significant predictor variables (Detached Protector and PDDS) and fatigue (FSMC) as an outcome variable are printed in gray. The model fit for the final model was excellent [Chi^2^ (5) = 4.58, *p* = 0.470, Chi^2^/*df* = 1.12, CFI > 0.99, RMSEA < 0.01]. Additionally, this model was confirmed with another fatigue measure [CFQ; Chi^2^ (5) = 7.31, *p* = 0.199, Chi^2^/*df* = 1.12, CFI = 0.99, RMSEA = 0.03]. Thus, whereas depression symptoms were not specifically predicted by one of the SMI dysfunctional coping modes, only the Detached Protector mode, representing detachment/avoidance as a coping strategy, predicted fatigue symptoms, irrespective of class assignment. Considering the magnitude of the standardized beta coefficients (0.33 for disability and 0.34 for the Detached Protector) it must be emphasized that psychological factors in terms of detached/avoidant coping make the same contribution to fatigue symptoms than reported physical symptoms. Replicating the model with the CFQ fatigue measure instead of the FSMC, even demonstrated that the standardized effect for the coping mode is more than twice as large than reported physical disability (0.16 for disability and 0.35 for the Detached Protector).

### The Relation Between Childhood Trauma, Alexithymia and the Detached Protector Mode

Finally, to explain the variance of the Detached Protector mode, the influence of the CTQ childhood trauma subscales and alexithymia on this mode were analyzed. This analysis was performed *post-hoc*, following the results obtained by Pust et al. (13) on the relation between childhood maltreatment, maladaptive schemas and fatigue symptoms. A linear regression analysis with the CTQ subscales and the TAS-26 as predictors and the Detached Protector mode as dependent variable was calculated. The final regression model is displayed in Table 4. Only the influence of CTQ emotional abuse, CTQ emotional neglect and the TAS-26 on the dependent variable Detached Protector mode were significant. Forty-four percentage of the Detached Protector mode's variance could be explained by the final model [*F*_(6, 564)_ = 75.15, *p* < 0.001, *f*^2^ = 0.79, (1–β) > 0.99]. The maximal value of the Variance Inflation Factor (VIF) was 2.39, allowing the neglect of multicollinearity. The residuals did not deviate significantly from the normal distribution (Kolmogorov–Smirnov, *p* > 0.999). Neither was there a noteworthy influence of outliers (maximal Cook's *d* = 0.05). Thus, the calculated regression analysis had an excellent fit. The results indicate that among different early adversities, only childhood emotional neglect and emotional abuse are associated by the development of later detached/avoidant coping as a significant predictor of fatigue symptoms. A significantly larger effect was even obtained for the impact of alexithymia on the Detached Protector mode.

**Table 4:** Results of the regression analysis on the relation between childhood maltreatment, alexithymia and the SMI detached protector mode.

	**Detached Protector Mode**	
	**β**	***p***
CTQ_S1_ emotional abuse	**0.14**	**0.005**
CTQ_S2_ physical abuse	−0.02	0.614
CTQ_S3_ sexual abuse	−0.03	0.318
CTQ_S4_ emotional neglect	**0.17**	**<0.001**
CTQ_S5_ physical neglect	< -0.01	0.947
TAS_26_total_score	**0.56**	**<0.001**
Model	*F*_(6, 564)_ = 75.15,	**<0.001**
	$R_{\text{adj}}^{2}$ = 0.44	

*Standardized regression coefficients are displayed. Significant values are printed bold*.

## Discussion

The present study aimed to provide a new psychosomatic conversion perspective on MS-fatigue. It combined a psychodynamic perspective on traumatic conversion with data-driven quantitative methods, utilizing the schema therapeutic mode model as an integrative conceptual framework. In a first step, PwMS were distinguished by their characteristic maladaptive coping strategies. These coping strategies represented the PwMS responses to adverse emotional states and can be distinguished into surrender, avoidance, and overcompensation. Latent profile analysis reveals three distinct groups of (1) PwMS with low overall maladaptive coping, (2) PwMS with heightened avoidance/surrender coping modes, and (3) PwMS with heightened avoidance/overcompensation coping modes.

On a somatic level, the three groups did not differ in their self-reported physical disability, measured with the PDDS. Thus, the presence of maladaptive coping in PwMS doesn't seem to be associated with difference in perceived somatic disability. There were also no statistically significant differences in other clinical or socio-demographic variables, such as disease course, medication or age. However, both groups of PwMS with heightened maladaptive coping modes also reported significantly higher fatigue as well as depression symptoms than PwMS with low overall maladaptive coping. This means that those PwMS that generally tend to utilize maladaptive coping strategies in order to deal with inner negative and stressful emotional states are also those individuals that report more subjectively experienced fatigue and depression symptoms.

This first step was however not yet sufficient to draw conclusions on a specific relation between maladaptive coping modes and fatigue or depression symptoms that might reflect a functional property of MS-fatigue. A subsequent path model was computed. To disentangle depression from fatigue, both constructs served as dependent variables. The BDI-FastScreen was used to capture the non-somatic features of depression only. A noticeable pattern was observed: depression and fatigue symptoms were correlated as widely described in the scientific literature (45). For depression symptoms, there was no specific relation with any of the maladaptive coping modes. This means that an individual that generally tends to resort to any maladaptive coping strategy also experiences heightened levels of depression. Moreover, the subjectively perceived somatic disability was not associated with depression. Contrary, fatigue was specifically predicted by the detached/avoidant coping mode. Moreover, the magnitude of this latter effect was similar and apparently even higher than the effect of physical disability on fatigue symptoms. This result speaks for a highly specific functional relation between detached/avoidant coping behavior, subjectively perceived disability, and fatigue. In combination with the aforementioned results, it indicates on the one hand that increased perceived somatic disability is one factor that is associated with MS-fatigue, irrespective of maladaptive coping, stressing a somatic link. On the other hand, detached/avoidant coping reflects a psychological mechanism that is linked to MS-fatigue.

In a last step, regression analysis was utilized to predict the Detached Protector mode from adverse childhood experiences and alexithymia. A high specificity was obtained for the impact of early emotional abuse and neglect, as well as alexithymia. This result is in line with the publication by Pust et al. (13) that focused on specific trait characteristics of PwMS with fatigue. Taken together, it can be hypothesized, based on the present results, that fatigue might reflect one facet of detached/avoidant coping that is distinct to depression and selectively related to adverse emotional childhood experiences. How can these results be transferred to the asymbolic trauma model of conversion?

The results of the present study can be interpreted in line with the trauma model of conversion as follows: The results suggest that emotional childhood adversities have elicited overwhelming negative effects in the early life course of PwMS, which have also been perceived in terms of bodily reactions and somatic excitation. However, due to a yet unspecified inability to process the excitation in the psychological realm, the individual tends to resort to splitting as an - in a broader sense -avoiding as well as protecting defense mechanism, being unconscious (46), and detachment/avoidance as the corresponding coping mechanism in line with the schema-therapeutic mode model (as indicated by the conscious Detached Protector mode). At a certain point, either prior or during the MS-disease, the overwhelming part of emotional excitation is discharged into the body and manifests the bodily symptom of fatigue. This corresponds to the somatic manifestation of the Detached Protector mode as a general coping strategy. In addition, the large effect size obtained for the relation between alexithymia and the Detached Protector mode supports the theoretical considerations made by psychoanalysts such as Sifneos (47) and Nemiah (48), stating that alexithymic patients would only perceive the physical qualities of their emotions. PwMS with detached/avoidant coping modes tend to be detached to their emotional experiences. However, they report high levels of perceived fatigue, although they do not differ in their subjective disability from those PwMS who demonstrate low levels of maladaptive coping and low levels of fatigue. It is plausible, in line with the work of Pirlot and Corcos (49), that alexithymia as an early resistance to overwhelming emotions shares functional properties with the Detached Protector mode as a coping mechanism to overwhelming emotions, and fatigue, as the potential bodily manifestation of it. Its association with childhood adversities also supports the view that the Detached Protector mode develops as a coping mechanism dealing with adverse emotions. It is noteworthy, that emotionally adverse experiences in particular seem to recruit these processes.

Since the present study suggests an asymbolic trauma-related perspective on MS-fatigue, future research could also aim at establishing a link between the psychoneurotic conversion model and MS-fatigue, or even linking it to Janet's understanding of dissociation. In accordance with the view of a symbolic conflictual conversion, fatigue would symbolize both, (1) the early trauma/conflict, indicated by emotional abuse/neglect (“my primary caregivers were so exhausted that they couldn't adequately fulfill my needs”), and (2) the plea trying to mobilize the environment (“I'm exhausted, I feel underserved”) (50). From a psychoanalytical view, fatigue could also be seen as a symptom that arises from a desire (for example for attachment) and the moral prohibition on realizing this desire (51). In the first, traumatic case of the conversion model, fatigue would like an emotional exhaustion, following traumatic injuries. In the second, conflictual case, fatigue would be the symbolization of an emotional blockade. The CTQ would then represent the adverse inter-subjective emotion regulation disorder, whereas the SMI would represent the symbolization of this disorder. However, to investigate the appropriateness of the symbolic conversion model to explain the etiology and nature of MS-fatigue by means of an empirical study, it would be mandatory to also assess unconscious phenomena, such as the individual's conflicts or defense mechanisms. The study could be extended by applying measures, such as the Defense Style Questionnaire (52) or the OPD structure questionnaire (53). However, it also has to be considered that the assessment of psychodynamic constructs by means of an online assessment might face limitations too. Additional interviews by clinical experts could serve to evaluate the validity of online data. For the application of the dissociation model, it can only be speculated, whether or not an individual could recall childhood adversities in an online assessment, if these experiences are dissociated.

The study also faces some limitations: one key limitation of the present study is that all data were obtained cross-sectionally and thus are correlational in nature. The schema-therapeutic mode model implies that maladaptive coping modes originate in early childhood and manifest in later life. However, there might exist a recall-bias for certain childhood adversities in individuals with different maladaptive coping modes. Additionally, for the evaluation of the external validity of the present study, it has to be considered that data was gathered web-based. The clinical data as well as the socio-demographic data -including disease course, medication, and presence of a psychiatric disorder- assessed in this study relies on PwMS' self-reports. A selection bias for the PwMS sample can also not be excluded. The high levels of fatigue in the present sample might result from the advertisement of the study that focused on fatigue. Thus, the external validity of the data might be particularly tailored to PwMS with fatigue. Likewise, depression scores were relatively high, lying above the clinical cut-off scores that are commonly found among PwMS (54). The use of structured clinical interviews would have helped to validate symptoms. As the data assessment was web-based, the participation of bed-ridden patients or patients who do not have access to the Internet might have been limited. Regarding the external validity of the applied measures, it has to be considered that the present study relied on a measure that was developed to assess schema modes but not psychodynamic constructs. Even if schema therapy has adopted psychodynamic theories and concepts, its validity to capture those constructs that are vital to study a conversion model might be limited. Mixed methods approaches could help to overcome potential limitations that derive from the assessment of more differentiated psychodynamic processes by means of standardized questionnaires.

Taking into account the strengths and limitations of the present study, it provides a first approximation to the understanding of MS-fatigue in accordance with the asymbolic trauma model of conversion. To the best of our knowledge, this is the first study that tried to take a psychodynamic perspective on MS-fatigue by means of a data-driven empirical approach. To overcome the limitations of the present study and broaden the understanding of fatigue as an at least partial conversion disorder, future studies should try to utilize expert interviews and include psychodynamic measures specifically.

Deepening the understanding of the psychological roots of MS-fatigue could help to inform its successful treatment as well as the application of psychotherapeutic interventions (55), or even the identification of PwMS that are susceptible to the development of fatigue due to certain etiological risk factors. It also has to be noted that with the introduction of DSM 5, the criterion of a psychogenic cause in conversion disorders has been deleted (16). However, the present study confirms that a psychogenic perspective on fatigue can have valuable implications for the understanding of its genesis. Consequently, keeping a conversion perspective on somatic disorders in both, practice and research, remains as topical as ever (56). Especially since embodiment entered neuroscience (57), the interaction of bodily states and emotions has received visibility also in the empirical sciences and the treatment of trauma-associated ill-health (58). Therefore, the obstacle of an underrepresentation of the conversion model in contemporary publications (59) must not distract from the numerous scientific publications that have proven the asset of the conversion models and the promising future avenues they still might provide.

## Data Availability Statement

The raw data supporting the conclusions of this article will be made available by the authors, without undue reservation.

## Ethics Statement

The studies involving human participants were reviewed and approved by University of Konstanz ethics review board, University of Konstanz, Germany. The patients/participants provided their written informed consent to participate in this study.

## Author Contributions

GP, RS, and SG designed the study. GP, CD, and JR were responsible for data collection. GP and RW conducted the statistical analysis and the data pre-processing. GP, RS, and LG wrote the manuscript. CD, JR, MK, RW, and BR critically revised the manuscript. All authors interpreted the results and gave critical feedback.

## Conflict of Interest

GP received speaker honoraria and project funding from Genzyme Sanofi and speaker honoraria from Novartis. CD has received honoraria from Novartis and Merck. RS reports speaker honoraria from Novartis. SG reports honoraria from Mylan GmbH, Almirall S. A., and Celgene and research grants from Biogen. The remaining authors declare that the research was conducted in the absence of any commercial or financial relationships that could be construed as a potential conflict of interest.
